# Mind the information gap: fertility rate and use of cesarean delivery and tocolytic hospitalizations in Taiwan

**DOI:** 10.1186/2191-1991-1-20

**Published:** 2011-12-12

**Authors:** Ke-Zong M Ma, Edward C Norton, Shoou-Yih D Lee

**Affiliations:** 1 Department of Healthcare Administration and Medical Informatics, Kaohsiung Medical University, Kaohsiung, Taiwan; 2 Department of Health Management and Policy, University of Michigan, Ann Arbor, MI, USA; 3 Department of Economics, University of Michigan, Ann Arbor, MI, USA

**Keywords:** information, physician inducement, cesarean delivery, fertility, tocolysis

## Abstract

**Background:**

Physician-induced demand (PID) is an important theory to test given the longstanding controversy surrounding it. Empirical health economists have been challenged to find natural experiments to test the theory because PID is tantamount to strong income effects. The data requirements are both a strong exogenous change in income and two types of treatment that are substitutes but have different net revenues. The theory implies that an exogenous fall in income would lead physicians to recoup their income by substituting a more expensive treatment for a less expensive treatment. This study takes advantages of the dramatic decline in the Taiwanese fertility rate to examine whether an exogenous and negative income shock to obstetricians and gynecologists (ob/gyns) affected the use of c-sections, which has a higher reimbursement rate than vaginal delivery under Taiwan's National Health Insurance system during the study period, and tocolytic hospitalizations.

**Methods:**

The primary data were obtained from the 1996 to 2004 National Health Insurance Research Database in Taiwan. We hypothesized that a negative income shock to ob/gyns would cause them to provide more c-sections and tocolytic hospitalizations to less medically-informed pregnant women. Multinomial probit and probit models were estimated and the marginal effects of the interaction term were conducted to estimate the impacts of ob/gyn to birth ratio and the information gap.

**Results:**

Our results showed that a decline in fertility did not lead ob/gyns to supply more c-sections to less medically-informed pregnant women, and that during fertility decline ob/gyns may supply more tocolytic hospitalizations to compensate their income loss, regardless of pregnant women's access to health information.

**Conclusion:**

The exogenous decline in the Taiwanese fertility rate and the use of detailed medical information and demographic attributes of pregnant women allowed us to avoid the endogeneity problem that threatened the validity of prior research. They also provide more accurate estimates of PID.

JEL Classification: I10, I19, C23, C25

## Background

Since Kenneth Arrow's seminal article in 1963,[1] health economists have been interested in information asymmetry in the health care market. The physician-induced demand (PID) hypothesis is essentially that physicians engage in some persuasive activity to shift the patient's demand curve in or out according to the physician's self interest. Patients have incomplete information about their condition and may be vulnerable to this advertising-like activity [2]. McGuire and Pauly [3] developed a general model of physician behavior that emphasized PID was tantamount to strong income effects. Empirical health economists have been challenged to find natural experiments to test the theory. The data requirements are both a strong exogenous change in income and two types of treatment that are substitutes but have different net revenues. The theory implies that an exogenous fall in income would lead physicians to recoup their income by substituting a more expensive treatment for a less expensive treatment. Given the longstanding controversy surrounding PID, this is an important theory to test.

Drawing on McGuire and Pauly's model, Gruber and Owings [4] hypothesized that an income effect should lead obstetricians and gynecologists (ob/gyns) to induce demand for the more lucrative cesarean sections (c-sections) over vaginal deliveries. They tested the hypothesis with data in the U.S and found that a 10 percent fertility drop corresponded to an increase of 0.6 percentage points in the probability of undergoing a c-section. McGuire,[2] however, pointed out this result did not preclude other income-recovery effects. Omitting the existence of cesarean delivery on maternal request (CDMR) may also make the interpretation of their results ambiguous. Lo [5] provided a detailed review on the relationship between financial incentive and c-section use, indicating that the empirical evidence is mixed. Moreover, some studies reviewed in Lo's paper have relied on regional samples, samples from selected hospitals or patient subpopulations, or samples lacking the required clinical information, and these limitations would lead to a doubtful interpretation of their findings.

An important modification of the basic hypothesis is that the extent of inducement depends on the extent of the asymmetric information between physicians and patients [1,6]. Patients who are relatively less informed are more likely to be induced. Well-informed patients are not. This extension places an additional burden on the empirical data-identifying well-informed patients. The basic premise of physician-induced demand is that physicians may exploit the information gap between themselves and their patients. If so, PID should be more likely where the information gap is greater [7-9]. Physicians themselves, presumably, are informed health consumers and should be knowledgeable about the health risks and benefits associated with different methods of delivery. Similarly, female relatives of physicians have low cost of obtaining reliable medical information [10]. Chou et al. [10] found that female physicians and female relatives of physicians were significantly less likely to undergo a c-section than other high socioeconomic status (SES) women. The definition of health information gap in their study may be questionable, however. The household registry used in the study could only be linked to those women co-residing with physicians, thus potentially misclassifying into the comparison group relatives of physicians who, although living in a different household, may be equally informed of the relative benefits and risks of c-sections versus vaginal deliveries. This misclassification may lead to underestimation of the true difference in the c-section use between physicians' relatives and other women. The use of occupation as the only criteria in the classification was also problematic. Highly educated women could be medically informed irrespective of their occupation, but they were included in the non-medically-informed group in Chou et al.'s study [10].

In the absence of a gold standard to measure health information gap, examining women's choice of the delivery mode by SES may be useful in empirical testing of the physician-induced demand hypothesis. Several studies have analyzed the relationship between SES and mother's preference for vaginal deliveries versus c-sections, and they all showed a significant association between women's high level of SES and low preference of surgical delivery [11-15]. These findings all imply that education and SES play an important role in women's decisions about the delivery mode and could serve as a good proxy to measure of the health information gap.

In this study, we empirically examine McGuire and Pauly's [3] PID hypothesis and its extension based on c-sections in Taiwan because this medical procedure and recent demographic changes in Taiwan provide the requisite variation for an empirical testing of the hypothesis. A rapid decline in the fertility rate in Taiwan has led to falling income for ob/gyns. If the PID hypothesis is valid, ob/gyns have at least two strategies to recoup the lost income. First, to the extent possible, they could substitute c-section for vaginal delivery because c-section has a much higher reimbursement rate. Second, they could encourage the use of other expensive medical procedures, notably inpatient tocolysis, to make up for the income loss in deliveries. We also expand on what Chou et al. [10] did in their study by also exploring the potential difference between high and low SES women. Compared to their low SES counterparts, high SES women may be more medically informed but were included in the non-medically-informed group in the study.

## Methods

### Data

The primary data source is Taiwan's National Health Insurance Research Database (NHIRD) that consists of comprehensive longitudinal use and enrollment history of all National Health Insuance (NHI) beneficiaries in Taiwan. This study combines the following NHIRD datasets spanning from 1996 to 2004: registry for contracted medical facilities, registry for medical personnel, registry for contracted beds, registry for beneficiaries, registry for board-certified specialists, hospital discharge file, and registry for catastrophic illness patients. Data on fertility and population size are obtained from the 1996-2004 Taiwan-Fuchien Demographic Fact Book. These data were merged with the NHI claims data by the area codes. Vaginal deliveries and c-sections are both paid under a prospective payment system (PPS) according to a patient's principal discharge diagnosis or based on the principal operative procedures as defined by the International Classification of Diseases, Ninth Revision, Clinical Modification (ICD-9-CM). During the period of our study, the rates of reimbursement were higher for c-sections than for vaginal deliveries; CDMR was reimbursed at the cost of a vaginal delivery and the woman had to pay the difference to the provider. The NHI reimbursement scheme for delivery is provided in Table 1.

**Table 1:** Reimbursement Scheme of Deliveries by NHI

Accreditation status	Reimbursements for c-section	Reimbursements for vaginal delivery and CDMR (YYYY/MM/DD)^a^
Medical center	NT$ 31,500 (1997/10/01~1998/06/30)	NT$ 17,000 (1995/05/01~1998/06/30)
	NT$ 32,330 (1998//07/01~2001/05/31)	NT$ 17,420 (1998/07/01~2001/05/31)
	NT$ 33,280 (2001/06/01~2004/06/30)	NT$ 17,910 (2001/06/01~2004/06/30)
	NT$ 33,969 (2004/07/01~2005/12/31)	NT$ 18,268 (2004/07/01~2005/04/30)
	NT$ 36,086 (2006/01/01~)	NT$ 33,969 (2005/05/01~2005/12/31)
		NT$ 36,086 (2006/01-01~)
Regional hospital	NT$ 30,000 (1997/10/01~1998//06/30)	NT$ 16,000 (1995/05/01~1998/06/30)
	NT$ 30,740 (1998/07/01~2001/05/31)	NT$ 16,370 (1998/07/01~2001/05/31)
	NT$ 31,480 (2001/06/01~2004/06/30)	NT$ 16,760 (2001/06/01~2004/06/30)
	NT$ 32,169 (2004/07/01~2005/12/31)	NT$ 17,118 (2004/07/01~2005/04/30)
	NT$ 34,286 (2006/01/01~)	NT$ 32,169 (2005/05/01~2005/12/31)
		NT$ 34,286 (2006/01/01~)
District hospital	NT$ 28,500 (1997/10/01~1998//06/30)	NT$ 15,000 (1995/05/01~1997/02/28)
	NT$ 29,230 (1998/07/01~2001/05/31)	NT$ 15,500 (1998/03/01~1998/06/30)
	NT$ 29,600 (2001/06/01~2004/06/30)	NT$ 15,880 (1998/07/01~2001/05/31)
	NT$ 30,403 (2004/07/01~2005/12/31)	NT$ 16,070 (2001/06/01~2005/06/30)
	NT$ 32,520 (2006/01/01~)	NT$ 16,485 (2004/07/01~2005/04/30)
		NT$ 30,403 (2005/05/01~2005/12/31)
		NT$ 32,520 (2006/01/01~)
Clinic	NT$ 27,000 (1997/10/01~1998//06/30)	NT$ 14,000 (1995/05/01~1997/02/28)
	NT$ 27,170 (1998/07/01~2001/05/31)	NT$ 15,000 (1998/07/01~2001/05/31)
	NT$ 27,170 (2001/06/01~2004/06/30)	NT$ 15,100 (2001/06/01~2004/06/30)
	NT$ 27,319 (2004/07/01~2005/12/31)	NT$ 15188 (2004/07/01~2005/04/30)
	NT$ 29,436 (2006/01/01~)	NT$ 27,319 (2005/05/01~2005/12/31)
		NT$ 29,436 (2006/01/01~)

a dates (YYYY/MM/DD) are in parentheses.

In addition to providing more c-sections, ob/gyns may recoup their income loss from a decline in fertility by encouraging the use of other expensive medical services. In this study, we focus on tocolytic hospitalizations. Among on/gyn inpatient services, tocolysis is closely related to the conditions that accompany the decline in fertility observed in Taiwan--i.e., late marriage, older childbearing age, and increased use of artificial reproductive technology and services. Several studies have reported that antenatal hospitalization with pregnancy-related diagnosis represents a significant health and economic burden for women of reproductive age [16-18]. One of the most common causes for antenatal hospitalizations is symptoms due to preterm labor and is often treated with tocolytic therapy [19]. However, the effectiveness of inpatient tocolysis for preterm labor remains unclear and no guideline for the appropriate use exists, leaving the treatment at the physician's discretion [19-21]. An interesting fact to note in Taiwan is that the use of inpatient tocolysis has remained relatively stable while the number of newborns has declined significantly. These trends raise the possibility that ob/gyns may induce the use of inpatient tocolysis to recoup the income loss due to the decline in fertility.

### Study Population and Operational Definitions of Delivery Modes and Inpatient Tocolysis

This study population included all singleton deliveries between 1996 and 2004. Based on the NHI diagnosis-related groups (DRG) codes in NHI hospital discharge files, we categorized delivery modes as vaginal delivery (DRG = 0373A), c-section (DRG = 0371A), and CDMR (DRG = 0373B, maternal request c-section and no ICD-9 conditions required). The NHI in Taiwan paid the full cost of a c-section if the delivery mode was medically indicated. If the c-section was not medically indicated, then the patient must pay out of pocket. Due to this regulation, doctors, if at all possible, would classify a c-section as medically indicated for the financial benefit of the patient. Therefore, we could be reasonably sure that those c-sections classified as CDMR (DRG = 0373B) were in fact not medically indicated. Ob/gyns, clinics, and hospitals may up-code clinical complications to help patients seek full reimbursement for c-sections. To the extent up-coding existed, the number of CDMR would be under-reported and our estimation of the effect of fertility decline on CDMR would be conservative. To prevent up-coding, the Bureau of National Health Insurance (BNHI) exercised close oversight and imposed a severe financial penalty on transgressions. Fines for fraud were 100 times the amount of the false claim charged to the BNHI [22,23]. We believe that the coding system was quite accurate because the government regularly audited claims and because of the fines [23]. To make this study comparable to previous research, the following exclusion criteria were employed: women above 50 and below 15 years of age, attending ob/gyn's age below 25 and above 75, and women whose deliveries involved more than one child (ICD-9-CM 651.0 to 651.93). In total, 2,241,980 singleton deliveries in Taiwan between 1996 and 2004 were identified and analyzed.

To identify the use of inpatient tocolysis, we first excluded early pregnancy loss and induced abortion from the hospital discharge file. We then followed a recent study by Coleman et al. [21] to define inpatient tocolytic hospitalization as having one of the following ICD-9-CM codes: 644.00, 644.03, 644.10, and 644.13. In the hospital discharge file, each patient record had one principal diagnosis, as listed in the ICD-9-CM, and up to four secondary diagnoses. We identified tocolytic hospitalization from the primary and secondary diagnosis. Following Coleman et al.'s approach,[21] we further excluded women contraindicated for tocolysis according to the current standard of care and women noted to have additional medical conditions that could have been treated with medications misclassified with tocolysis, because these conditions required either immediate c-section or termination of pregnancy, including ICD-9-CM codes 642, 762.0, 762.1, 762.2, 761, 656.3, 663.0, 768.3, 768.4, 762.7, and 740-759. Based on these definitions, a total of 96,838 tocolytic hospitalizations were identified.

### Main Explanatory Variables

Our empirical approach was built on prior work,[4,24] with a twist of incorporating the general fertility rate (GFR) as an aggregate measure of women's preference for the delivery mode and the number of ob/gyns per 100 births as an indication of PID. Women's preference for c-sections and physician-induced demand both predict that a falling fertility rate will lead to increased c-section and tocolytic hospitalization use. However, women's preference for c-sections is only related to fertility decline whereas physician-induced demand operates through the *ratio *of ob/gyns to births and the decision belongs largely to ob/gyns. This distinction allowed us to have an empirical approach that could measure each effect independently. Specifically, we hypothesized that a decline in the general fertility rate would increase the probability of having a CDMR, ceteris paribus, because low fertility would increase the social value of newborns and increase women's preference for c-sections over vaginal deliveries. An increase in ob/gyns per 100 births, on the other hand, would increase the probability of women having a c-section or tocolytic hospitalization on less informed women, ceteris paribus, because ob/gyns per 100 births measure negative income shock to ob/gyns. In other words, the coefficient on the general fertility rate would capture the effect of fertility decline on women's preference of the delivery mode, holding constant ob/gyns per 100 births, and the coefficient is expected to be negative; the marginal effect of the interaction term "ob/gyns per 100 births*information", holding constant the general fertility rate, is an estimate of PID and is expected to be positive.

Considering the dynamics of ob/gyns market entry or exit, the variable ob/gyns per 100 births may not be a perfect measure of ob/gyn financial pressure. Because a physician's decision to start a practice depends on market conditions, identification of financial pressure solely by ob/gyn density may cause bias and inconsistency [2,25]. Thus, we used the one-year lagged number of ob/gyns per 100 births instead of the number of ob/gyns per 100 births. The lagged number of ob/gyns per 100 births should be highly correlated with the number of ob/gyns, but was unlikely to be correlated with unmeasured demand factors. This would reduce the reverse causality problem in the results.

The other main explanatory variable was GFR, an age-adjusted birth rate, defined as: GFR = [number of live births/females aged 15-49] × 1000. The specification improved previous estimations by taking the demographic composition into consideration.

Because this study aimed to compare the likelihood of choosing a delivery mode and having a tocolytic hospitalization between medically-informed individuals versus other women, the specification of health information gap was critical. We measure the information gap using a combination of two approaches. The first approach, which followed prior research,[10,26] differentiated female physicians and female relatives of physicians from other women. We identified female physicians by matching the anonymous identifiers of eligible women listed on the NHI enrollment files against the medical personnel registry. Female relatives of physicians were operationalized as those living in the same household of a physician and were identified by using the NHI enrollment files. There were 3,038 female physicians (0.13% of all observations), 57,999 female relatives of physicians (2.59% of all observations), and 2,180,943 other women (97.27% of all observations) in our study population.

The second approach used monthly insurable wage to classify women into three SES groups. Monthly insurable wage was calculated based on the woman's wage, if she was the insured or the head of the household, or based on wage of the household head, if she was a dependent. The NHI program is financed by wage-based premiums from people with clearly-defined monthly wage and fixed premiums from those without a clearly-defined monthly wage. Women with a clearly-defined monthly insurable wage were assigned to one of the three SES categories: (1) high SES, women with monthly insurable wage greater than or equal to NT$40,000 (≧ US$1,280), (2) middle SES, women with monthly insurable wage between NT$39,999 and NT$20,000 (US$1,280 and US$640), and (3) low SES, women with monthly insurable wage less than NT$20,000 (< US$640). Women without clearly-identified monthly wage were assigned to the low SES group; they included farmers, fishermen, the low-income, and subjects enrolled by the district administrative offices (Chen et al., 2007; Chou, Chou, Lee, and Huang, 2008). Based on this definition, we identified 189,349 high SES women (8.45% of all observations), 426,320 middle SES women (15.63%) and 1,626,311 low SES women (72.54%). Using insurable wage to measure pregnant women's SES has been employed in several studies in Taiwan,[10,26,27] and the percentage of low SES women in our sample statistics was quite close to those in prior reports.

### Other covariates

We assumed that the choice of the delivery mode would also be influenced by clinical and non-clinical factors [28]. Clinical factors included previous c-section, fetal distress, dystocia, breech, and other complications. Non-clinical individual-level variables included woman's age and insurable wage. Non-clinical institutional factors included ownership (public, private non-profit, or proprietary), teaching status (teaching or non-teaching institution), accreditation status (medical center, regional hospital, district hospital, or ob/gyn clinics), and hospital bed size [29]. Ob/gyn factors included the attending ob/gyn's age and gender. Because patient parity was not available in the data set, we adopted a standard ICD-9-based classification to code complications into mutually exclusive categories, including previous c-section (ICD-9-CM 654.2), fetal distress (ICD-9-CM 656.3, 663.0, 768.3, and 768.4), dystocia (ICD-9-CM 652.0, 652.3-652.4, 652.6-652.9, 653, 659.0, 659.1, 660, 661.0-661.2, 661.4, 661.9, and 662), breech (ICD-9-CM 652.2 and 669.6), and other complications (ICD-9-CM 430-434, 641, 642, 647.6, 648.0, 648.8, 654.6, 654.7, 655.0, 656.1, 656.5, 658.1, 658.4, and 670-676).

For the test of the effects of inducement and information gap on tocolytic hospitalization, we controlled for physician, institutional, and individual factors in addition to log of lagged ob/gyn per 100 births and log GFR following a prior study by Ma et al. [30]. Physician characteristics included attending obstetrician/gynecologist's age and gender. The attending ob/gyn's years in the specialty were not included because it was highly correlated with age. Institutional factors included hospital ownership, teaching status, accreditation status, and bed size. Individual factors included the woman's age, wage, having prior pregnancy-associated hospitalizations (ICD-9-CM codes from 640 to 676 with a fifth digit of "0" or "3", or any diagnosis in combination with a code V22 (normal pregnancy) or V23 (high-risk pregnancy)), having a major disease card, and the previous year's inpatient expenses. Having a major disease card was an indicator of having a severe health problem such as malignant neoplasm, end-stage renal disease, chronic psychotic disorder, cirrhosis of the liver, acquired immunodeficiency syndrome, and schizophrenia.

### Sample statistics

Table 2 shows the trends of fertility and singleton deliveries by modes in Taiwan from 1996 to 2004. Overall, there are 773,768 (32.75%) cases of c-sections (including CDMR) among 2,280,487 singleton deliveries. The national c-section (including CDMR) rate increased slightly from 30.87% in 1996 to 31.92% in 2004. Notably, the rate of CDMR was 0.80% in 1996 and it peaked at 2.74% in 2002, whereas the GFR dropped from 54 in 1996 to 34 in 2004. Table 3 showed the decrease in the average revenue from singleton deliveries among ob/gyns, confirming that the decline in fertility did cause negative income shock to ob/gyns. The number of ob/gyns, hospitals, and clinics reduced substantially from 1996 to 2004. The average revenue from singleton deliveries among ob/gyns was affected much more than that of hospitals and clinics, confirming that the declined fertility did cause negative income shock to ob/gyns. The revenues from tocolytic hospitalizations increased over time, supporting our expectation that health care providers may induce more tocolytic hospitalizations to recoup their income loss due to the rapid fertility decline.

**Table 2:** Trends of Fertility and Delivery Modes in Taiwan, 1996 to 2007

Year	General fertility rate	Number of births	Number of vaginal deliveries (%)	Number of c-sections (%)	Number of CDMR (%)
1996	54	324,317	201,767 (73.72%)	69,520 (25.40%)	2,412 (0.88%)
1997	53	324,980	201,080 (67.42%)	93,139 (31.23%)	4,025 (1.35%)
1998	43	268,881	161,206 (65.75%)	79,695 (32.51%)	4,256 (1.74%)
1999	45	284,073	169,141 (66.01%)	82,674 (32.27%)	4,406 (1.72%)
2000	48	307,200	181,020 (65.68%)	88,989 (32.29%)	5,588 (2.03%)
2001	41	257,866	157,067 (65.84%)	75,753 (31.75%)	5,753 (2.41%)
2002	39	246,758	152,168 (65.81%)	73,268 (31.69%)	5,780 (2.50%)
2003	36	227,447	143,675 (66.67%)	66,956 (31.07%)	4,855 (2.25%)
2004	34	217,685	140,638 (67.68%)	63,498 (30.56%)	3,651 (1.76%)
2005	33	206,462	133,275 (73.83%)	43,999 (24.37%)	3,245 (1.80%)
2006	33	205,720	131,225 (73.27%)	44,057 (24.60%)	3,801 (2.13%)
2007	32	203,711	128,225 (72.39%)	44,664 (25.21%)	4,244 (2.40%)

Total	NA	2,463,343	1,900,487 (68.40%)	826,212 (29.73%)	52,016 (1.87%)

*Note*.

1. General fertility rates were obtained from http://sowf.moi.gov.tw/stat/year/y02-04.xls

2. Number of births was obtained from http://www.ris.gov.tw/ch4/static/yhs609700.xls

Numbers in column 4 to 6 were calculated from 1996 to 2007 NHIRD where vaginal delivery is defined by DRG code 0373A, c-section is defined by DRG code 0371A, and CDMR is defined by DRG code 0373B.

**Table 3:** The Effect of Declining Fertility on Ob/gyns' Revenue^a^

Year	Number of attending ob/gyns	Average number of singleton deliveries performed	Average revenue from singleton deliveries (in NT$)	Average revenue from inpatient tocolysis (in NT$)
1996	1,879	177.22	3,343,926.08	148,431.73
1997	1,685	186.43	3,653,196.72	157,001.29
1998	1,666	153.58	3,088,646.87	142,946.03
1999	1,657	159.92	3,244,554.32	158,192.13
2000	1,614	172.50	3,504,260.61	165,691.29
2001	1,625	144.14	2,958,485.39^a^	152,658.26^a^
2002	1,614	137.25	2,864,625.75^a^	157,025.88^a^
2003	1,594	134.95	2,992,693.05^a^	154,092.17^a^
2004	1,587	135.66	3,062,313.78^a^	182,177.66^a^

Total	3,044	NA	NA	

a Due to the implementation of global budgeting in 2001, those revenues are the points of worth for singleton deliveries and inpatient tocolysis from 2001 to 2004, and they need to be adjusted by the dollar value per service point. So the actual revenues will be lower than the numbers listed.

As Table 4 shows, there were 693,492 medically-indicated c-sections (30.93% of all singleton deliveries), and 40,726 CDMR (1.82% of all singleton deliveries). The average age to give birth was 28.15, and the average age of undergoing c-section was older than that of vaginal delivery. The sample for the information gap analysis contained 3,038 births (0.14%) born to female physicians, 57,999 births (2.59%) born to female relatives of physicians, and 2,182,943 births (97.27%) born to other women; 189,349 births (8.45%) were born to high SES women, 426,320 births (15.63%) to middle SES women, and 1,626,311 births (75.92%) to low SES women. Physicians and physicians' relatives had lower crude CDMR rates (1.67% and 1.19%, respectively) than other women (2.93%). Interestingly, high SES women had a higher c-section and CDMR rate (2.39%) than middle and low SES women (1.98% and 1.74%, respectively). However, these were crude rates, without adjustment for complications. The most striking difference between the c-section and vaginal delivery columns was having a previous c-section. Among all vaginal delivery cases, only 0.41% had a previous c-section. Nearly 14% of all c-section cases (including CDMR) had a previous c-section, and this rate was close to the rates reported in other studies using the NHIRD in Taiwan [10,22,27,31].

**Table 4:** Summary Statistics of Patients by Delivery Modes, 1996-2004^a^

Variables	All births	Vaginal delivery (DRG = 0373A)	C-section (DRG = 0371A)	CDMR (DRG = 0373B)
***Social-demographic variables***				
Age (S.D.)	28.15 (4.86)	27.55 (4.73)	29.63 (4.81)	29.07 (5.16)
Wage (S.D.)	17229.22 (16301.26)	17071.82 (16182.48)	17353.54 (16350.62)	17947.48 (17446.45)
Female physicians (%)	3,038 (0.14%)	1,967 (67.00%)	920 (31.34%)	49 (1.67%)
Female relatives of physicians (%)	57,999 (2.59%)	41,525 (72.74%)	14,879 (26.07%)	679 (1.19%)
Other women (%)	2,180,943 (97.27%)	1,409,325 (64.62%)	719,493 (32.99%)	52,125 (2.39%)
High SES women (%)	189,349 (8.45%)	124,257 (65.62%)	60,984 (32.21%)	4,108 (2.17%)
Low SES women (%)	1,626,311 (75.92%)	1,097,628 (67.49%)	500,320 (30.76%)	28,363 (1.74%)
Middle SES women (%)	426,320 (15.63%)	281,286 (65.98%)	136,593 (32.04%)	8,441 (1.98%)
***Institutional characteristics***				
Bed size (S.D.)	489.21 (756.45)	474.53 (741.26)	482.69 (755.18)	391.82 (658.89)
Ownership				
Public (%)	307,572 (13.72%)	203, 280 (13.48%)	100,074(14.43%)	4,218 (10.36%)
Private non-profit (%)	632,443 (28.21%)	430,669 (28.56%)	192,341 (27.74%)	9,433 (23.16%)
Proprietary (%)	1,301,965 (58.07%)	873,813 (57.96%)	401,077 (57.83%)	27,075 (66.48%)
Accreditation status				
Medical center (%)	311,422 (13.89%)	206,992 (13.73%)	98,912 (14.26%)	5,518 (13.55%)
Regional hospital (%)	484,075 (21.59%)	334,758 (22.20%)	142,808 (20.60%)	6,509 (15.98%)
District Hospital (%)	632,326 (28.20%)	419,879 (27.85%)	199,946 (28.83%)	12,501 (30.70%)
Clinic (%)	814,157 (36.32%)	546,133 (36.22%)	251,826 (36.31%)	16,198 (39.77%)
Teaching status				
Teaching (%)	987,515 (44.05%)	661,572 (43.88%)	309,998 (44.70%)	15,945 (39.15%)
Non-teaching (%)	1,254,465 (55.95%)	846,190 (56.12%)	383,494 (55.30%)	24,781 (60.85%)
***Ob/Gyn characteristics***				
Ob/Gyn Gender (S.D.)	0.94 (0.24)	0.93 (0.25)	0.94 (0.25)	0.95 (0.22)
(0 if female; 1 if male)	39.49 (1.88)	39.47 (1.88)	39.52 (1.91)	39.53 (1.74)
Ob/Gyn age (S.D.)	39.49 (1.88)	39.47 (1.88)	39.52 (1.91)	39.53 (1.74)
***Complications in c-section***				
Fetal distress (%)	54,670 (2.44%)	5,761 (0.38%)	48,276 (6.81%)	633 (1.55%)
Dystocia (%)	194,877 (8.69%)	15,430 (1.02%)	176,918 (25.51%)	2,529 (6.21%)
Breech (%)	136,817 (6.10%)	2,614 (0.17%)	133,516 (19.25%)	687 (1.69%)
Others (%)	203,273 (9.07%)	87,837 (5.83%)	112,592 (16.24%)	2,844 (6.98%)
Previous c-section (%)^b^	313,812 (14.00%)	6,197 (0.41%)	304,262 (43.87%)	3,353 (8.23%)

Observations	2,241,980	1,507,762	693,492	40,726

a Following Xirasagar and Lin (2007), and Liu, Chen, and Lin (2008), deliveries without a DRG code in NHIRD (totally 38,507 cases) were excluded in all analyses.

b History of previous c-section was reported only for women who had had more than one delivery.

### Research Hypotheses

The study tested three research hypotheses:

Hypothesis 1: Compared to their counterparts, women who were less medically-informed would be more likely to undergo c-sections as the ratio of ob/gyn to births increased.

Hypothesis 2: The exogenous decline in fertility (GFR) would also increase the use of CDMR, regardless of the women's access to medical information.

Hypothesis 3: Compared to their counterparts, women who were less medically-informed would be more likely to have inpatient tocolysis as the ratio of ob/gyn to births increased.

### Multinomial Probit Model on the Use of C-section and CDMR

We used multinomial probit model to test the first hypothesis. The basic model had a dependent variable with three discrete outcomes: c-section, vaginal delivery, and CDMR. These outcomes were mutually exclusive and not ranked. The multinomial probit model provides the most general framework to study discrete choice models because it allows correlation between all alternatives [32]. The indirect utility function that individual *i *choosing alternative *j *with ob/gyn *g *in hospital *h *in region *r *at time *t *can be written as:

(1) $$V_{ighrtj}=W_{ighrtj}^{\prime }\beta_{j}+\varepsilon_{ighrtj}$$

This specification results if we assume that *ε_ighrtj _*are identically normally distributed with covariance matrix Ω. Let *W *denote a set of explanatory variables $\left[\text{ln}\left(\text{OBBIRT}{\text{H}}_{\text{rt}}\right)\text{,Inf}{\text{o}}_{\text{ighrt}}\text{,ln}\left({\text{OBBIRTH}}_{\text{rt}}\right)\times \text{Inf}{\text{o}}_{\text{ighrt}}\text{,}\right)X_{ighrt},\ln\left(Fertility_{rt}\right),Z_{ghrt},H_{hrt},\left(\delta_{r},\varsigma_{t}\right]$, and *j *∈ {1, 2, 3}. *j *is the discrete choice of delivery mode (1 if vaginal delivery, 2 if c-section, 3 if CDMR), *i *indexes individual patient, *g *indexes ob/gyn, *h *indexes hospital, *r *indexes region, *t *indexes time, and *β *is the coefficient on the explanatory variables. *ln*(*Fertility_rt_*) is the log of region's GFR in region *r *in year *t*, and *ln*(*OBBIRTH_rt_*) is the log of the lag number of ob/gyns per 100 of birth in region *r *in year *t*. *Info_ighrt _*is an indication of being medically informed individual (i.e., *Info_ighrt _*= 1 indicates female physicians and female relative of physicians, or high SES women; *Info_ighrt _*= 0 indicates other women (compared to female physicians and female relative of physicians) or low SES women). A full set of regional and year dummies are also included to control for the regional fixed effects (*δ_r_*) and time fixed effects (*ζ_t_*), respectively. *X *is a vector of observable patients' characteristics, *Z *is a vector of observable ob/gyn characteristics, *H *is a vector of observable hospital characteristics.

The probability that patient *i *choosing alternative *j *with ob/gyn *g *in hospital *h *in region *r *at time *t *is then given by:

(2) $$P_{ighrt1}=\text{Pr}\left(Y_{ighrt}=1\right)=\int_{-\infty }^{\left(W_{igher1}-W_{igher2}\right)\beta }\int_{-\infty }^{\left(W_{ighrt1}-W_{ighrt3}\right)\beta }f\left(\varepsilon_{ighrt1}-\varepsilon_{ighrt2},\varepsilon_{ighrt1}-\varepsilon_{ighrt3}\right)d\left(\varepsilon_{ighrt1}-\varepsilon_{ighrt3}\right)d\left(\varepsilon_{ighrt1}-\varepsilon_{igher2}\right)$$

(3) $$P_{ighrt2}=Pr\left(Y_{ighrt}=2\right)=\int_{-\infty }^{\left(W_{igher2}-W_{igher1}\right)\beta }\int_{-\infty }^{\left(W_{ighrt2}-W_{ighrt3}\right)\beta }f\left(\varepsilon_{ighrt2}-\varepsilon_{ighrt1},\varepsilon_{ighrt2}-\varepsilon_{ighrt3}\right)d\left(\varepsilon_{igher2}-\varepsilon_{ighrt3}\right)d\left(\varepsilon_{ighrt2}-\varepsilon_{igher1}\right)$$

(4) $$P_{ighrt3}=1-Pr\left(Y_{ighrt}=1\right)-Pr\left(Y_{ighrt}=2\right)$$

where *f *is the bivariate normal density function.

Empirically, we took double difference from the multinomial probit models to get the marginal effects of the interaction terms and thereby answered the hypotheses [33,34]. More specifically, the marginal effect of the interaction term can be expressed as:

Inducement effect = $\left[{\hat{P}}_{\mathrm{OBBIRTH}2004,NI}-{\hat{P}}_{OBBIRTH1996,NI}\right]-\left[{\hat{P}}_{OBBIRTH2004,I}-{\hat{P}}_{OBBIRTH1996,I}\right]$

If the inducement hypothesis held, the inducement effect was expected to be positive and significant. We calculated the interaction effect using the average of the probabilities method. The method calculates the probability for each observation four times with changing the character of interest (i.e., log of lagged ob/gyn per 100 births and information status), and then get the interaction effect. The following expression is the interaction effect where the probability $\hat{P}$ is calculated with average log of lagged ob/gyn per 100 births in 2004 of informed patients minus $\hat{P}$ calculated with average log of lagged ob/gyn per 100 births in 1996 of informed patients:

$$\left[\begin{array}{cc} \left(\hat{\text{P}}\left(\text{ln}\left(OBBIRTH\right)=-0.291,Info=0\right)\right)\\ -\left(\hat{\text{P}}\left(\text{ln}\left(OBBIRTH\right)=-0.613,Info=0\right)\right) & \end{array}\right]-\left[\begin{array}{cc} \left(\hat{\text{P}}\left(\text{ln}\left(OBBIRTH\right)=-0.291,Info=1\right)\right)\\ -\left(\hat{\text{P}}\left(l\text{n}(OBBIRTH),=,-,0,.,613,,,I,n,f,o,=,1\right)\right) & \end{array}\right]$$

Finally, all above equations would be estimated with the Huber-White robust standard errors, in order to control for the heteroskedasticity in nonlinear models. Also, all equations would be estimated with the cluster option in STATA to adjust standard errors for intragroup correlation, and the cluster identifier was the highest level units of the model (i.e., hospital/clinic).

### Probit Models on the Use of Inpatient Tocolysis

We then used the probit model to estimate physician-induced inpatient tocolysis (hypothesis 3). The probability that patient *i *had a tocolytic hospitalization in hospital *h *in region *r *at time *t *was given by:

(5) PrYighrt=1=Φα+γ1 lnOBBIRTHrt+γ2Infoighrt+γ12 lnOBBIRTHrt×Infoighrt+(1)γ3 lnFertilityrt+β1Xighr+β2Zghrt+β3Hhrt+δr+ςt+μi+εighrt

where *ln*(*OBBIRTH_rt_*) is the log of lag ob/gyn per 100 births. *Info_ighrt _*is an indicator variable of being medically informed (female physicians and female relatives of physicians, or high socioeconomic status women). In equation (5), the main variable of interest was the interaction between the measures of supply and information gap. We also assumed that the probability of receiving tocolytic hospitalizations would be affected by *X*, *Z*, and *H*. *X *was a vector of observable patients' characteristics (including woman's age, insurable wage, having prior pregnancy-associated hospitalizations, having a major disease card, and previous year's inpatient expenses), and *X *thus captured the health conditions of pregnant women that increased the likelihood of tocolytic hospitalization. *Z *is a vector of observable ob/gyn characteristics (including attending ob/gyn's age and gender), and *H *is a vector of observable hospital characteristics (including hospital ownership, teaching status, accreditation status, and bed size).

With one continuous variable *ln*(*OBBIRTH_rt_*) and one dummy variable (*Info_ighrt_*) interacted in the above probit equation, the interaction effect is the discrete difference (with respect to *Info_ighrt_*) of the single derivative (with respect to *ln*(*OBBIRTH_rt_*). Formally,

(6) $$\begin{array}{llll} \frac{\Delta \frac{\partial E\left[Y_{ighrt}|ln\left(OBBIRTH_{rt}\right),Info_{ighrt},W\right]}{\partial ln\left(OBBIRTH_{rt}\right)}}{\Delta Info_{ighrt}} & =\left(\gamma_{1}+\gamma_{12}\right)\varphi \left(\left(\gamma_{1}+\gamma_{12}\right)ln\left(OBBIRTH_{rt}\right)+\gamma_{2}+W\beta \right)\; & & \;\\ & -\gamma_{1}\varphi \left(\gamma_{1}ln\left(OBBIRTH_{rt}\right)+W\beta \right)\; & & \;\\ & \; & \end{array}$$

where and $E\left[Y_{ighrt}|\ln\left(OBBIRTH_{rt}\right),Info_{ighrt},W\right]$ are the conditional means of the dichotomous dependent variable *Y_ighrt_*, *ϕ *is the probability density function of the standard normal distribution, and the vector *W *represents all exogenous right hand side variables. Clearly, the magnitude of the marginal effect is conditional on the value of the independent variables. The marginal effect of the interaction term thus captures the rapidly declining effect on the inducement of those who were less medically-informed individuals affected by the ob/gyns' inducement, relative to medically-informed individuals who were less likely to be affected by the ob/gyns' inducement behavior. If the inducement hypothesis held, the interaction effect was expected to be positive and significant. Unfortunately, the interaction effect was difficult to compute in STATA package due to the extremely large sample size in this study. We thus calculated the marginal effect of the interaction term using the average of the probabilities method. The method was to calculate the probability for each observation four times with changing the character of interest (i.e., log of lagged ob/gyn per 100 births and information status), and then recalculated the marginal effect interaction term.

## Results

### The Role of Information Gap and the Inducement Effects

Tables 5 and 6 are the empirical results from multinomial probit models with two different definitions of health information gap to test the inducement effect on c-section use. These findings show that the interaction effects "information × log of lagged ob/gyn per 100 births" were not statistically different from zero, i.e. the declining fertility rate did not increase the use of c-sections conditional on patients' professional background and presumed better access to health information. The empirical results suggest that the inducement effect on c-sections is approximately zero, and the standard errors are tight, so we can rule out an effect as small as 0.06 (the effect found in Gruber et al.'s study [4]). Hence, although decline in fertility would increase the income pressure on ob/gyns, it did not lead them to substitute the higher reimbursed c-sections. Moreover, even there was a significantly negative correlation between fertility and use of CDMR, the correlation did not vary by the presumed access to health information, on average. In other words, the results supported our research hypothesis 2 but not research hypothesis 1.

**Table 5:** Multinomial probit estimates of the effects of declining fertility and health information gap on c-section use (Base outcome: vaginal delivery; Treatment group: female physicians and female relatives of physicians; Comparison group: other women; Main explanatory variable: log of lagged ob/gyns per 100 births × Information), 1996-2004^a^

	**C-section**		**C-section on maternal request**	
Variables	Coef.	Robust Std. Err.	Coef.	Robust Std. Err.
Log of lagged ob/gyns per 100 births	0.174***	0.038	0.339***	0.091
Log of lagged ob/gyns per 100 births × Information^b^	-0.008	0.134	-0.293***	0.106
Information^b^	-0.304	0.164	-0.103*	0.057
Log GFR	-0.291	0.285	-0.681***	0.084
***Patients' characteristics***				
Age	0.056***	0.001	0.055***	0.002
Insurable wage (÷10^2^)	-0.0004***	0.00005	-0.0003***	0.0001
Previous c-section	7.503***	0.025	3.785***	0.038
Fetal distress	4.672***	0.018	---^c^	---^c^
Dystocia	4.598***	0.027	---^c^	---^c^
Breech	3.761***^e^	0.034	---^c^	---^c^
Other complications	4.517***	0.019	---^c^	---^c^
***Hospitals' characteristics***				
Private non-profit	-0.538***	0.021	0.195***	0.031
Proprietary	0.150***	0.028	1.175***	0.04
Medical Center	0.156***^e^	0.044	0.582***	0.059
Regional Hospital	-0.408***	0.031	0.123**	0.042
District Hospital	-0.158***	0.02	0.470***	0.023
Teaching Hospital	0.132***	0.027	0.081**	0.034
Bed size (÷10^2^)	-0.028***	0.002	-0.0002	0.002
***Ob/gyn characteristics***				
Ob/gyn age	0.006	0.01	0.002	0.013
Ob/gyn gender	0.091	0.067	0.152	0.084
Constant	-9.240**	2.66	-2.51	4.173

Log likelihood	-4,399,462.47			

a The regression includes a full set of time and regional dummies and *N *= 2,241,980.

b Information is a dummy variable and information = 1 indicates medically-informed individuals.

* Statistically significant at the 10% level.

** Statistically significant at the 5% level.

*** Statistically significant at the 1% level.

^c ^Coefficients and standard errors were not estimated because CDMR by definition does not have medical complications.

g The marginal effect of the interaction term "Log of lagged ob/gyn per 100 births × Information" on the probability of having c-sections:

$$\begin{array}{c} \left[\begin{array}{c} \left(\mathrm{Pr}\left(LOBBIRTH=-0.2910312,I=0\right)\right)\\ -\left(\mathrm{Pr}\left(LOBBIRTH=-0.6134288,I=0\right)\right) \end{array}\right]-\left[\begin{array}{c} \left(\mathrm{Pr}\left(LOBBIRTH=-0.2910312,I=1\right)\right)\\ -\left(\mathrm{Pr}\left(LOBBIRTH=-0.6134288,I=1\right)\right) \end{array}\right]\\ =0.0004363 \end{array}$$

Standard error for the marginal effect obtained by bootstrapping: 0.0005167

h The marginal effect of the interaction term "Log of lagged ob/gyn per 100 births × Information" on the probability of having CDMR:

$$\begin{array}{c} \left[\begin{array}{c} \left(\mathrm{Pr}\left(LOBBIRTH=-0.2910312,I=0\right)\right)\\ -\left(\mathrm{Pr}\left(LOBBIRTH=-0.6134288,I=0\right)\right) \end{array}\right]-\left[\begin{array}{c} \left(\mathrm{Pr}\left(LOBBIRTH=-0.2910312,I=1\right)\right)\\ -\left(\mathrm{Pr}\left(LOBBIRTH=-0.6134288,I=1\right)\right) \end{array}\right]\\ =0.0001728 \end{array}$$

Standard error for the marginal effect obtained by bootstrapping: 0.0006485

**Table 6:** Multinomial probit estimates of the effects of declining fertility and health information gap on c-section use (Base outcome: vaginal delivery; Comparison group: low socioeconomic status women; Treatment group: High socioeconomic status women; Main explanatory variable: log of lagged ob/gyns per 100 births × Information), 1996-2004^a^

	**C-section**		**C-section on maternal request**	
Variables	Coef.	Robust Std. Err.	Coef.	Robust Std. Err.
Log of lagged ob/gyns per 100 births	0.789**	0.35	0.591***	0.13
Log of lagged ob/gyns per 100 births × Information^b^	0.133	0.291	-0.054	0.39
Information^b^	-0.188	0.513	-1.746**	0.655
Log GFR	-0.207	0.231	-0.588**	0.089
***Patients' characteristics***				
Age	0.057***	0.001	0.055***	0.002
Insurable wage (÷10^2^)	-0.0004***	0.00005	-0.0005***	0.0001
Previous c-section	6.750***	0.029	3.322***	0.091
Fetal distress	5.467***	0.035	---^c^	---^c^
Dystocia	6.528***	0.045	--- ^c^	---^c^
Breech	3.784***	0.086	---^c^	---^c^
Other complications	4.529***	0.017	---^c^	---^c^
***Hospitals' characteristics***				
Private non-profit	-0.653***	0.061	0.139**	0.07
Proprietary	0.087	0.074	1.041***	0.094
Medical Center	0.332**	0.119	0.612***	0.137
Regional Hospital	-0.275***	0.075	0.263**	0.099
District Hospital	-0.088**	0.037	0.585***	0.047
Teaching Hospital	0.084	0.065	0.003	0.074
Bed size (÷10^2^)	-0.030***	0.005	0.003	0.005
***Ob/gyn characteristics***				
Ob/gyn age	-0.001	0.005	-0.011*	0.006
Ob/gyn gender	0.003	0.024	0.126***	0.034
Constant	-5.446***	0.19	-5.219***	0.262

Log likelihood	-4,160,195.98			

a The regression includes a full set of time and regional dummies and *N *= 1,815,660

b Information is a dummy variable and information = 1 indicates medically-informed individuals.

* Statistically significant at the 10% level.

** Statistically significant at the 5% level.

*** Statistically significant at the 1% level.

^c^Coefficients and standard errors were not estimated because CDMR by definition does not have medical complications.

The marginal effect of the interaction term "Log of lagged ob/gyn per 100 births × Information" on the probability of having c-sections:

$$\begin{array}{c} \left[\begin{array}{c} \left(\mathrm{Pr}\left(LOBBIRTH=-0.2910312,I=0\right)\right)\\ -\left(\mathrm{Pr}\left(LOBBIRTH=-0.6134288,I=0\right)\right) \end{array}\right]-\left[\begin{array}{c} \left(\mathrm{Pr}\left(LOBBIRTH=-0.2910312,I=1\right)\right)\\ -\left(\mathrm{Pr}\left(LOBBIRTH=-0.6134288,I=1\right)\right) \end{array}\right]\\ =0.0003987 \end{array}$$

Standard error for the marginal effect obtained by bootstrapping: 0.0006423

The marginal effect of the interaction term "Log of lagged ob/gyn per 100 births × Information" on the probability of having CDMR:

$$\begin{array}{c} \left[\begin{array}{c} \left(\mathrm{Pr}\left(LOBBIRTH=-0.2910312,I=0\right)\right)\\ -\left(\mathrm{Pr}\left(LOBBIRTH=-0.6134288,I=0\right)\right) \end{array}\right]-\left[\begin{array}{c} \left(\mathrm{Pr}\left(LOBBIRTH=-0.2910312,I=1\right)\right)\\ -\left(\mathrm{Pr}\left(LOBBIRTH=-0.6134288,I=1\right)\right) \end{array}\right]\\ =0.0002126 \end{array}$$

Standard error for the marginal effect obtained by bootstrapping: 0.0007081

According to the results from the multinomial probit model, several other explanatory variables such as women's age, insurable wage, having previous c-sections, having maternal complications (e.g., fetal distress), hospital bed size, hospital accreditation status (non-clinic), private non-profit ownership, proprietary ownership, and teaching hospital were significantly associated with the likelihood of having c-section. These variables were also significantly associated with the likelihood of having CDMR, except for maternal complications and bed size.

### Test of the Spillover Effect on Inpatient Tocolysis

Table 7 shows the empirical results from probit models with two different definitions of health information gap to test the inducement effect on inpatient tocolysis. Again, the interaction effects are not statistically different from zero, suggesting that decline in the fertility rate did not lead ob/gyns to supply more tocolytic hospitalizations to less medically-informed patients, ceteris paribus. However, the positive coefficients on the log of lagged ob/gyn per 100 births implies that the higher ratio of ob/gyn per 100 births, the more tocolytic hospitalizations will be provided (see Table 7). Therefore, ob/gyns may supply more tocolytic hospitalizations to compensate their income loss, regardless of pregnant women's access to health information.

**Table 7:** Probit estimates for equation (5): the effects of declining fertility and health information gap on the probability of having tocolytic hospitalizations, 1997-2004 (Base outcome: having no tocolytic hospitalizations)^a^

	**Specification 1**		**Specification 2**	
	(Treatment group: female physicians and female relatives of physicians; Comparison group: other women)		(Treatment group: high socioeconomic status women; Comparison group: low socioeconomic status women;)	
Variables	Coef.	Robust Std. Err.	Coef.	**Robust Std. Err**.
Log of lagged ob/gyn per 100 births	0.174***	0.038	0.339***	0.091
Log of lagged ob/gyn per 100 births × Information^b^	-0.008	0.134	-0.293	0.206
Information^b^	-0.103*	0.057	-0.304	0.164
Log GFR	0.966	0.681	-1.127	0.81
***Patients' characteristics***				
Age	0.027***	0.001	0.025***	0.001
Insurable wage (÷10^2^)	-0.0002***	0.0002	0.0003	0.0002
Having a major disease card	0.016	0.018	0.012	0.049
Having pregnancy-associated hospitalizations before	0.521***	0.006	0.693***	0.009
Previous year's inpatient expenses	0.0001***	0.0002	0.0001***	0.0001
***Hospitals' characteristics***				
Public	-0.155***	0.01	-0.187***	0.025
Private non-profit	-0.214***	0.108	-0.403*	0.196
Medical center	0.127	0.219	0.188	0.254
Regional Hospital	0.113***	0.012	0.050***	0.002
District Hospital	0.045***	0.007	0.100***	0.015
Teaching Hospital	0.068***	0.011	0.048*	0.027
Bed size (÷10^2^)	-0.007***	0.001	-0.007	0.002
***Ob/gyn characteristics***				
Ob/gyn age	-0.002	0.002	0.002	0.004
Ob/gyn gender	0.020*	0.01	0.066**	0.029
Constant	-2.390***	0.079	-8.413***	0.16

Number of observations	1,941,935		1,770,654	

Log likelihood	-181,362.22		-199,483.47	

a The regression includes a full set of time and regional dummies.

b Information is a dummy variable and information = 1 indicates medically-informed individuals.

* Statistically significant at the 10% level.

** Statistically significant at the 5% level.

*** Statistically significant at the 1% level.

The marginal effect of the interaction term "Log of lagged ob/gyn per 100 births × Information" on the probability of having toocolytic hospitalizations (for specification 1):

$$\begin{array}{c} \left[\begin{array}{c} \left(\mathrm{Pr}\left(LOBBIRTH=-0.2910312,I=0\right)\right)\\ -\left(\mathrm{Pr}\left(LOBBIRTH=-0.6134288,I=0\right)\right) \end{array}\right]-\left[\begin{array}{c} \left(\mathrm{Pr}\left(LOBBIRTH=-0.2910312,I=1\right)\right)\\ -\left(\mathrm{Pr}\left(LOBBIRTH=-0.6134288,I=1\right)\right) \end{array}\right]\\ =0.0001165 \end{array}$$

Standard error for the marginal effect obtained by bootstrapping: 0.0005519

The marginal effect of the interaction term "Log of lagged ob/gyn per 100 births × Information" on the probability of having toocolytic hospitalizations (for specification 2):

$$\begin{array}{c} \left[\begin{array}{c} \left(\mathrm{Pr}\left(LOBBIRTH=-0.2910312,I=0\right)\right)\\ -\left(\mathrm{Pr}\left(LOBBIRTH=-0.6134288,I=0\right)\right) \end{array}\right]-\left[\begin{array}{c} \left(\mathrm{Pr}\left(LOBBIRTH=-0.2910312,I=1\right)\right)\\ -\left(\mathrm{Pr}\left(LOBBIRTH=-0.6134288,I=1\right)\right) \end{array}\right]\\ =0.0001728 \end{array}$$

Standard error for the marginal effect obtained by bootstrapping: 0.0004824

Compared to clinics, patients in regional or district hospitals were more likely to have tocolytic hospitalizations, because the turn-over rate of inpatient tocolysis is much lower than other ob/gyn inpatient procedures, they may tend to refer patients who needs tocolystic hospitalization to regional or district hospitals, which often have more empty beds than medical centers. Note that our results indicate that teaching hospitals are more responsive to income loss (in terms of inpatient tocolysis) than non-teaching ones. A possible explanation is that high-risk deliveries may have much better outcomes when they are transferred to a tertiary-level hospital (e.g., teaching hospital) with a high volume of obstetric and neonatal services,[35] and many district and regional hospitals in Taiwan are also teaching hospitals [36]. Finally, most ob/gyn clinics do not have enough ob/gyns on staff and better infrastructure to deal with complicated maternal and neonatal problems.

Furthermore, it has been discussed in previous literature that proprietary providers may respond more aggressively than private non-profit or public providers to the financial incentives [37]. Our analysis showed that holding other variables constant, patients had a lower probability to receive tocolytic hospitalizations in public and private non-profit providers compared to patients treated in proprietary hospitals. This finding is consistent with theoretical predictions and prior studies [30]. To our knowledge, most private providers are ob/gyn clinics in Taiwan, and providing tocolytic hospitalizations could be one of the strategies to recoup their income loss due to declined fertility.

## Discussion

Our study builds and improves upon the existing literature in several ways. First, our study expands the scope of extant literature and improves our understanding of PID in a different health care system. Second, analyzing data from a national dataset with comprehensive clinical information across all providers and patients means that there is no selection bias. The large number of observations provides great statistical power. Third, we can identify medically informed individuals two different ways (i.e., female physicians, female relatives of physicians, and high SES women) and then compare the propensity of undergoing c-section (including CDMR) and having tocolytic hospitalizations of these individuals versus other women. Fourth, we can control for another possible explanation for changes in the c-section rate by controlling for c-sections attributable to CDMR. Research is limited on this issue because data on CDMR are not readily identifiable in most clinical or national databases [38]. With information on CDMR, we would also be able to examine whether increased c-section use is a result of PID or, alternatively, change in women's preference. Finally, in contrast to the multiple-payers structure in the U.S. health care system, where most extant PID research was conducted, the universal health insurance and the single-payer system in Taiwan offer a favorable research setting that prevents the use of cumbersome methods to control for variation and change in health insurance coverage.

Although this study did not find a statistically significant inducement effect on the use of c-sections under the rapid declining fertility rate, some ob/gyns appeared to have recouped their income loss by supplying more tocolytic treatments. To the extent that a change in the physician's return from inducement (e.g., fertility goes down) stimulates a change in influence (more inpatient tocolysis supplied), this study provides some evidence for the PID hypothesis. A possible explanation for the insignificant inducement effect on the use of c-sections is that a c-section is fairly inexpensive relative to other medical technologies,[4] so when facing rapidly declining fertility rate, ob/gyns can supply other medical procedures that are more lucrative than c-sections.

With regard to the role of the health information gap, the empirical findings did not support the hypothesis that less medically-informed women preferred more c-sections than vaginal deliveries when the fertility rate was low. Nevertheless, given the existence of asymmetric information between providers and patients, it may be argued that physicians would be likely to induce service use. Therefore, investigating the degree to which physician inducement occurs, rather than whether inducement exists, is perhaps a more fruitful direction for further investigation [39].

An interesting finding in this study was that the declining fertility rate increased the use of CDMR. There are two possible explanations. First, women may be more likely to have CDMR when the fertility rate goes down because they believe that c-sections are safer and more beneficial for the baby, and the tremendous importance of having a healthy baby given the low fertility rate provides much of the impetus for having a c-section [40]. Second, cultural beliefs and practices influence the perception and desire about labor and delivery mode and several studies have reported that the desire to have a child born on an auspicious date and time may be one major reason for CDMR in Taiwan [41,42]. If the fertility rate continues to decline, it is plausible that parents would be more inclined to request c-sections at an auspicious time in order to bestow their baby a bright future and to bring harmony to both the family and the baby [43]. Future research may also collect primary data to explain why the rate of CDMR increases as the fertility rate declined.

There are several limitations in our study and these limitations could motivate future research. First, our measures of patients' access to health information were constrained by data availability. The two indicators may not accurately reflect health information access and may affect the validity of the findings. Besides c-section and tocolysis treatment, ob/gyns may employ other strategies to recover income loss due to fertility decline. Provision of artificial reproductive services and consultation is an example. An ideal measure of the income effect is the share of an ob/gyn's total practice income (including both inpatient and outpatient revenues as well as other services not covered by NHI) that is derived from delivery procedures. Ignoring other practice revenues may underestimate the effect of other possible income-recovery strategies. Furthermore, our study used the mean of patients' age and the proportion of patients with major disease card as adjustments for patient's disease severity. More precise case-mix adjustment should be considered when comparing different providers' practice in future research.

Several additional methodological caveats are worth noting. First, this study lacked data on parity and birth weight, which may affect the choice of delivery mode [44]. Second, we were unable to explicitly account for some physician and institutional factors, such as physician's demand for leisure, tax benefits, and hospital/clinic staffing constrains,[45-47] which may confound the findings. Third, the use of disaggregated data in the analyses of tocolytic care may ignore patients' demand factors for tocolytic hospitalizations. For instance, the increased use of assisted reproductive technology, postponement of marriage and childbearing ages, as well as an increasing number of low-weight and preterm births may also explain the increasing trend of the use of inpatient tocolysis. Patients' demand factors, such as increasing female labor supply and better education among women, may also affect women's fertility decision in Taiwan. Moreover, air pollution has also increased the number of low birth weight and premature infants in Taiwan,[48] and may contribute to the increasing use of tocolytic hospitalization. Future research (e.g., longitudinal analyses on soociodemographic structure change, fertility decision, and health care use) will be needed to disentangle the effects of PID on health care use and to inform policies.

Finally, although we used two different ways to identify the information gap (i.e., female physicians, female relatives of physicians, and high SES women) and obtained consistent findings, our study may still suffer from potential endogeneity bias - the effects of a decline in fertility may not be comparable across the treatment and control groups. Future research should take this issue into consideration before drawing any definitive causal conclusions.

## Conclusions

Findings from this study also raise some critical issues. First, it sheds light on what determines maternal and ob/gyns' choices of delivery modes during a period of dramatically declining fertility. This study also offers a precautionary note to countries where privatization of health care and its financing is ushering in ingenious ways of cost containment. The disproportionately high c-section rates in Taiwan may also hold major lessons for the many countries contemplating or having universal health insurance coverage with a similar mix of providers.

From the policy point of view, our results on the inducement of inpatient tocolysis use also raise concerns about the effect of payment reform on obstetric deliveries. Many countries have attempted different ways to contain the continuing increase in c-section rates, such as health education and peer evaluation, external review, public dissemination of c-section rates, medical malpractice reform, and changes in physician and hospital reimbursement,[49-53] and these strategies differ in their assumptions regarding their feasibility and the determinants of physicians' autonomy [42]. Among these strategies, changes in physician and hospital reimbursement draw the most attentions because health service researchers cite financial incentives as a major explanation for the growth of c-sections [54-57]. Higher fees for c-section are sometimes given as one explanation for the relatively high rates in many countries [5,58,59]. Because the costs to the obstetricians are similar on average for vaginal and c-section deliveries,[60] many have argued that equal fees might be preferable to the traditionally higher payments for c-sections [61,62]. Since results from this study do not support the hypothesis that ob/gyns would use more profitable c-sections to replace vaginal deliveries, the effectiveness of the c-section payment reform in Taiwan is yet to be determined. Policymakers should also be aware of the remarkable potential that decoupling physician reimbursement levels from the cost of the technology that is used may help to restrain the diffusions of procedures whose additional benefit is exceeded by their incremental cost. Countries with large or universally insured population should evaluate delivery profiles associated with the availability of health information, institutional size, and reimbursement policies. Future study could focus on the welfare implication associated with different delivery modes under rapidly declined fertility.

## List of abbreviations

PID: Physician-induced demand; Ob/gyns: Obstetricians/gynecologists; C-section: Cesarean sections; CDMR: Cesarean delivery on maternal request; SES: Socioeconomic status; NHI: National Health Insurance; NHIRD: National Health Insurance Research Database; PPS: Prospective payment system; ICD-9-CM: International classification of diseases, ninth revision, clinical modification; DRG: Diagnosis-related groups; BNHI: Bureau of National Health Insurance; GFR: General fertility rate; IV: Instrument variable.

## Competing interests

The authors declare that they have no competing interests.

## Authors' contributions

Ke-Zong Ma proposed the study, participated in data preparation and analyses, and drafted the manuscript. Edward C. Norton and Shoou-Yih Lee participated in the study design and helped to draft the manuscript. All authors read and approved the final manuscript.
